# Current Proposed Mechanisms of Action of Intravenous Immunoglobulins in Inflammatory Neuropathies

**DOI:** 10.2174/157015909790031166

**Published:** 2009-12

**Authors:** Saiju Jacob, Yusuf A Rajabally

**Affiliations:** Neuromuscular Clinic, Department of Neurology, University Hospitals of Leicester, United Kingdom

**Keywords:** Intravenous immunoglobulins, mechanisms of action, inflammatory neuropathy, Guillain Barre syndrome, Chronic inflammatory demyelinating polyneuropathy, multifocal motor neuropathy.

## Abstract

Intravenous immunoglobulins (IVIg) have been shown in a number of trials, to be an effective treatment for the three main types of inflammatory neuropathies: Guillain-Barré Syndrome (GBS), chronic inflammatory demyelinating polyneuropathy (CIDP), and multifocal motor neuropathy (MMN). IVIg is thought to exert its immunomodulatory effects by affecting several components of the immune system including B-cells, T-cells, macrophages, complement, cytokines and cellular adhesion molecules. This article reviews the published evidence and the principal postulated mechanisms of action of intravenous immunoglobulins with special emphasis on inflammatory neuropathies.

## INTRODUCTION

Intravenous immunoglobulins (IVIg) have been shown to be effective in a number of disorders with an underlying autoimmune basis. Amongst those, the inflammatory neuropathies, Guillain-Barré Syndrome (GBS), chronic inflammatory demyelinating polyneuropathy (CIDP), and multifocal motor neuropathy (MMN), have all been found in well-conducted randomised controlled trials (RCTs) to benefit from IVIg.

GBS is an acute polyradiculoneuropathy which could be demyelinating (as in the “AIDP”, i.e. “Acute Inflammatory Demyelinating Polyneuropathy” variant) or axonal (as in the “AMAN”, i.e. “Acute Motor Axonal Neuropathy” variant). Its incidence is estimated at 1 to 2 per 100,000. Respiratory involvement is common, and mortality is estimated at about 10%. Proximal and distal symmetric weakness with sensory symptoms but few sensory signs occur in the majority cases. Facial and bulbar weakness is not uncommon. Diagnosis is clinical but also relies on electrophysiology which may show signs of demyelination, and spinal fluid analysis typically shows a raised protein level with normal cellularity. The syndrome occurs in a post-infectious context in 75% of cases and probably results from cross-reactivity with the infectious agent and peripheral nerve sharing common epitopes to which autoantibody attack is directed. The most common infectious agent resulting in GBS is *Campylobacter jejuni*. Plasma exchanges were shown to be more effective than placebo for GBS in RCTs [50]. IVIg was subsequently found to be of equivalent effectiveness to plasma exchange [27] and has become standard treatment for GBS because of ease of administration and a comparatively better side-effect profile.

CIDP is an acquired heterogeneous disorder affecting sensory and motor peripheral nerves caused by a patchy demyelinating process, producing sensory loss and positive sensory symptoms as well as motor weakness. Its prevalence is of 3 to 5 per 100,000 and incidence of about 0.50 per 100,000/year. In its typical form, the disorder is symmetric and involves both proximal and distal limb regions. There are rarer atypical forms which can produce predominantly uni- or multifocal as well as distal involvement. Diagnosis relies on clinical features, and also mainly on electrophysiology, which allows demonstration of a demyelinating process, producing slowing of nerve conductions in various segments as well as conduction block. Cerebrospinal fluid protein level is raised in the majority of cases, and peripheral nerve histology, may show a demyelinating process with inflammatory features. Magnetic resonance imaging (MRI) can show thickened hyperintense nerve roots, trunks or plexi, consistent with the diagnosis. IVIg as well as steroids and plasma exchanges have been shown to be effective treatments for CIDP. The pathogenesis of CIDP is inflammatory, most probably autoimmune, involving both T cells and antibodies. The efficacy of IVIg in CIDP has been shown in different studies [26, 42].

MMN is a rare disorder with an estimated prevalence of 1 to 2 per 100,000 frequently occurring in middle-aged men. It predominantly affects the upper limbs with asymmetric weakness occurring in individual nerve distributions. There is typically no sensory involvement. The diagnosis is clinical and confirmed in the majority of cases by electrophysiology which shows evidence of motor conduction block. Anti-GM1 antibody positivity can be present in 30 to 80 % of cases. IVIg is the only treatment so far, found effective in MMN [4, 19, 36, 66].

## HISTORY OF USE OF INTRAVENOUS IMMUNOGLOBULIN

The two main components of the adaptive immunity are B-cells (which produce immunoglobulins) and T-cells (responsible for cell-mediated immunity). Immune deficiencies occurring in either of the components have been shown to increase susceptibility to bacterial, viral or fungal infections. Antibody therapy had been recognised as a useful tool since the late 19^th^ century when von Behring described antibodies against diphtheria. Replacement of immunoglobulins, either subcutaneously or intravenously, has been described as an effective therapy for primary immunodeficiencies for almost 50 years [9, 10]. In 1981 it was observed that intravenous immunoglobulin (IVIg) therapy in children with hypogammaglobulinemia and coincidental idiopathic thrombocytopenic purpura, led to a reproducible increase in platelet count [29]. This led way to the use of IVIg in several other diseases with a confirmed or suspected autoimmune aetiology.

Multiple sclerosis was one of the first neurological disorders in which IVIg was used [56], although it is not currently recommended by many guidelines. Since then, several reports of the successful use of IVIg have been published in several neuromuscular diseases including myasthenia gravis (MG) [1, 16, 18], CIDP [72], Gullain-Barre syndrome [2, 32] and multifocal motor neuropathy [46].

Currently, the use of IVIg can be classified broadly into low dose replacement therapy (usually 0.3-0.5g/kg every 3-4 weeks) or high-dose therapy for the anti-inflammatory action (usually 1-3g/kg). The comparison of IVIg dosing of 1.2g/kg over 3 days or 2.4 g/kg over 6 days in GBS has shown a better outcome for the six-day regimen [49]. However in CIDP lower initiating and maintenance doses may be sufficient [48], although large scale randomised trials looking into the exact dosing requirement are lacking. The half-life of IgG in the circulation is approximately 4 weeks, thereby requiring repeated courses every 8-12 weeks.

## MECHANISMS OF ACTION OF IVIg

Although the presence of “natural” antibodies capable of recognising foreign antigens could plausibly explain the role of IVIg in IgG replacement therapy, the precise mechanism of action by which IVIg exerts its immunomodulatory effects is not clearly understood. In inflammatory neuropathies there are several proposed pathophysiological mechanisms and a detailed review of these is beyond the scope of this article and has been dealt with elsewhere [25, 65, 67]. Pathological studies of nerve biopsies in CIDP and GBS reveal lymphocytic and macrophagic infiltrates in the endoneurium with deposits of IgM and complement components. The role of B-cells is clearly established in GBS where anti-ganglioside antibodies and complement activation have been demonstrated [67]. Lymphocytic infiltrates are predominantly T-cells recruited by chemokines and endothelial cell adhesion molecules. T-cells secrete matrix metalloproteases which break down endoneurial proteins. Macrophages are the predominant antigen presenting cells as demonstrated by increased expression of NFκB and the inflammatory cytokines, IL-6 and IL-1β. All these mechanisms are potentially modulated by IVIg and will be discussed in detail below. Paradoxically, plasma exchange which works theoretically opposite to IVIg by removing IgG from the body, seem to work in similar clinical situations.

### 1. Effect of IVIg on B-Cells and Antibodies

B-cells, which form 5-15% of the circulating lymphoid pool, are responsible for humoral immunity and acts against extracellular pathogens. B-cells are activated in response to a variety of stimuli and differentiate to form plasma cells. Plasma cells are usually restricted to the secondary lymphoid organs, comprising less than 0.1% of the lymphocytes in circulation. Autoreactive B-cells may be stimulated by either autoantigens or through non-specific polyclonal activation. Soluble immunoglobulins, produced by the plasma cells against autoantigens, are responsible for the majority of clinical features in antibody mediated autoimmune diseases. 

The use of IVIg in acquired haemophilia has shown that the presence of anti-idiotype antibodies against the anti-haemophilic factor (factor VIII antibodies) leads to a therapeutic suppression of these autoantibodies [53, 61]. Anti-idiotypes against autoantibodies to thyroglobulin [17], ANCA [51], acetylcholine receptor [77], DNA [58], platelet glycoproteien IIb/IIIa [41], beta-2 glycoprotein-I [7] and intrinsic factor [52] have been shown to be present in IVIg preparations. The presence of anti-idiotypes prevents the binding of pathogenic autoantibodies to the target epitopes, thereby ameliorating the autoimmune symptoms. Anti-idiotype antibodies are likely to be involved in the therapeutic effects of IVIg in GBS and MMN, where antibodies against different gangliosides have been described.

Other B-cell mediated effects of IVIg include inhibition of antibody production [33], inhibition of B-cell differentiation [60], inhibition of production of interleukin-6 and tumour necrosis factor-α [63], induction of B-cell apoptosis [64], down-regulation of specific auto-reactive B-cells [70] and regulation of B-cell subsets expressing CD5 [69], thereby suppressing the auto-antibody producing CD20+ B1 cells. Even though the serum anti-GM1 antibody levels in seropositive MMN patients do not fall after IVIg infusion, the Fab portion of Ig in the IVIg has been shown to inhibit binding of anti-GM1 Ab to target antigens [39]. Although the efficacy of IVIg in IgM paraproteinemic neuropathies is not well established, the levels of IgM myelin-associated glycoprotein (MAG) and sulfoglucuronyl paragloboside (SGPG) antibodies are reduced in occasional patients treated with this therapy [15].

FcRn (so named because it was initially identified in neonatal intestinal epithelium) is a protective receptor crucial for regulating the half life of IgG. In normal circumstances, IgG binds to FcRn and is protected from catabolism after being internalised in the endosome. IVIg is thought to saturate these receptors and thus accelerate the breakdown of endogenous IgG which may be mediating the autoimmune repertoire [76]. IVIg has also been shown in experimental animals to modulate B-cell migration from bone marrow to secondary lymphoid organs [62]. 

### 2. Effect of IVIg on T-Cells

T-cells mediate the cell mediated immunity and are involved in the handling of intracellular pathogens, but also play a major role in the regulation of B-cell responses. This is mediated by the two sub-population of CD4+ T-cells or T-helper cells (Th cells): Th1 cells secreting interleukin-2 (IL-2) and interferon-γ (IFN-γ) protect against intracellular pathogens and Th2 cells secreting IL-4, IL-5, IL-6 and IL-10 are effective in stimulating B-cells for antibody production. Th cells which normally fail to recognise auto-antigens can be primed by cross-reacting microbial molecules to recognise autoantigens in organ specific epithelial cells to induce autoimmune disease.

In animal models of experimental autoimmune encephalomyelitis and autoimmune uveitis, IVIg is thought to reduce the production of interleukin-2 and interferon-γ by T-cells [47, 55], preventing the development of disease. This is also thought to be one of the major effector mechanisms in the treatment of GBS and CIDP. IVIg preparations have also been shown to contain antibodies against CD4 cells [28], soluble HLA class I and II molecules [21], chemokine receptor, CCR-5 [8] and T-cell receptor β chain [40]. The possibility that therapeutic doses of immunoglobulins restores the balance between Th1 and Th2 cells has been the main rationale for several IVIg trials in multiple sclerosis [31].

### 3. Effect of IVIg on the Complement System

The heat-labile component of serum that augmented its bactericidal properties is one of the main pathogenic pathways involved in antibody-mediated autoimmune diseases. The formation of immune complexes activate the classical complement cascade resulting in the production of membrane attack complexes (MAC) which are thought to induce organ specific tissue damage in a variety of autoimmune diseases, like myasthenia gravis, lupus nephritis and GBS.

The anti-inflammatory activity of IVIg is at least partly mediated by its ability to prevent the formation of MAC and subsequent tissue destruction. Antibodies against several components of the classical complement pathway have been identified in IVIg. They include antibodies against C1 [43], C3a [6], C3b or C4 [20]. In addition, high doses of IVIg are thought to enhance the degradation of C3b [38]. In-vitro increase of complement uptake has been demonstrated in GBS and MG [5] and complement is thought to be important in GBS and its variant, Miller-Fisher syndrome [22, 23, 74], in which complement therapies are under investigation [22, 23].

### 4. IVIg Mediated Fc Receptor Blockade on Macrophages

FcγR receptors on the surface of macrophages can mediate inflammatory pathways by activating (FcγRI or FcγRIII) or inhibiting (FcγRII) different receptors [31]. IVIg may inhibit the FcγRI or FcγRIII receptors or upregulate the FcγRII receptors [14]. In idiopathic thrombocytopenic purpura (ITP), IVIg is thought to inhibit platelet phagocytosis through the FcγRII receptor [54]. In GBS and CIDP, inhibition of macrophage function reduces the phagocytosis of antigen-presenting cells and antibody-mediated cellular cytotoxicity, thus inhibiting macrophage-mediated demyelination [13]. Similarly an increase in ratio of FcγRII/FcγRIII receptors on monocytes have been demonstrated a week after IVIg administration in patients with CIDP and MMN who were beginning to improve [11].

### 5. Effect of IVIg on Cytokines

Cytokines are proteins or glycoproteins involved in the signalling process during a variety of immune reactions. Dysregulation of the cytokine system has been proposed as one of the mechanisms of autoimmunity. 

IVIg reduces the levels of circulating IL-1β in patients with GBS [57] and Kawasaki disease [37]. Thousand fold increase in the levels of IL-1 receptor antagonist have been shown after IVIg therapy [3]. However, cytokine modulation is unlikely to be the major mechanism of action of immunoglobulins, since IVIg remains functionally active in mice strains deficient in IL-1R, IL-4, IL-10, IFN-γR, IL-12β and TNF-α [12]. TNF-α mediated cytotoxicity is also inhibited by IVIg [59].

### 6. Effect of IVIg in Modulating Cell Migration

Leukocyte migration across biological barriers has been suggested to be an important mechanism in the causation of organ specific autoimmune diseases. IVIg is thought to modulate endothelial cell function by interacting with intercellular adhesion molecules (ICAM) [75]. A significant reduction in expression of ICAM-1 was seen in 8 out of 10 patients with MMN and CIDP, during the first week after infusion of IVIg [11].

Other possible mechanisms by which IVIg modulate cell migration include the presence of antibodies against integrins [34] and the argine-glycine-aspargine (RGD) cell adhesion motifs [71]. Inhibition of the chemokine (C-C motif) receptor-5 (CCR-5) by IVIg prevents the entry of HIV into its target cells [8]. 

### 7. Effect of IVIg on Superantigens

Superantigens like bacterial enterotoxins and viruses stimulate the Vβ chains of the T-cell receptor triggering the production of cytokines and breaking immune tolerance. The role of IVIG against the β-chains of the T-cell receptor [40] may thus be relevant in the influence of relapses triggered by infections in MG and CIDP [14].

### 8. Other Mechanisms of Action of IVIg

In toxic epidermal necrolysis, apoptosis of keratinocytes is prevented by blocking CD95, the Fas death receptor [73].

There is considerable variation in the sugar moiety attached to the aspargine 297 (N297) residue of the Fc portion of IgG. It has been suggested that sialic acid rich IgG levels are reduced in acute phases of several autoimmune disease models. Infusion of IVIg, which is pooled from several donors, may restore the level of sialic acid-rich IgG, thus inducing an anti-inflammatory action, possibly through novel receptors in regulatory macrophages. [30, 44, 45].

In addition to the above mentioned immune mediated effects, whether IVIg has a direct effect on remyelination [68] in diseases like GBS and CIDP is not very clearly understood.

## MECHANISMS OF SIDE-EFFECTS PRODUCED BY IVIG

Generally the side-effects of IVIg are usually minor occurring in less than 1 in 10 patients. Myalgia, chills or chest pain may occur in the first hour of infusion, which can be reversed by temporarily stopping the infusion and restarting at a slower rate. Fatigue, nausea and headache may occur for up to 24 hours. The exact pathogenesis of these side-effects are not clearly understood, but activation of the complement pathway by aggregated immunoglobulins may be partly responsible. This may also be partly responsible for the infrequent side-effect of aseptic meningitis.

Increase in serum viscosity produced by IVIg leads to increased risk of thrombo-embolic events, especially in pre-existing hyperviscosity syndromes like high cholesterol levels, hypergammaglobulinemia or cryoglobulinemia. Similarly, rate of infusion has to be slowed in patients with fluid overload like congestive cardiac failure. Although theoretically possible, hemolysis due to anti-A/B IgG blood group antibodies is almost never seen in clinical practice.

Severe anaphylactic reactions are fortunately rare, but cause a potential problem in selective IgA deficiency, which occurs in 1:1000 patients. These patients may have anti-IgA antibodies, which cross-react with the small IgA content in the infused IVIg, causing macromolecular complexes. Unless in an emergency situation, it is a good practice to check the immunoglobulin levels prior to commencement of IVIg therapy.

### Subcutaneous Immunoglobulin (SCIg) as an Alternative to IVIg

Side effects of IVIg are likely to be secondary to an allergic reaction to a large quantity of foreign protein. The administration of Ig by subcutaneous route (usually by a small portable pump at home) has been used mainly to avoid these systemic side effects, but also to reduce the cost and to maintain higher trough levels. SCIg has generally been employed in the treatment of primary immunodeficiencies. Some studies even suggest safer administration of SCIg in patients with IgA sensitisation and in pregnancy. SCIg has been shown to be effective in small series of patients with MMN[24] and CIDP [35]. However, SCIg has to be administered more frequently, can have local skin reactions and is contraindicated in patients on anticoagulant therapy and those with bleeding disorders and thrombocytopenia. Further large trials are required before this can be used as standard practice in inflammatory neuropathies.

**Table:** 

Most Plausible Mechanisms of Action of IVIg in Inflammatory Neuropathies
Anti-idiotype antibody production Inhibition of complement pathway Fc receptor modulation on macrophages and other effector cells Suppression of pathogenic cytokines Effects on cell migration by modulation of adhesion molecules T-cell modulation Direct effect on remyelination

## SUMMARY

Several immune components are likely to be important in the pathogenesis of GBS, CIDP and MMN. Demyelination in GBS and CIDP may happen along the course of the nerve, which requires a breakdown of blood-nerve barrier, where T-cells may be important for the pathophysiology. Demyelination also occurs distally in the intramuscular part of the nerve, where the barrier is absent, possibly mediated by humoral factors. Early in the disease course in GBS, T-cells may play an important role in pathogenesis as evidenced by increase in soluble IL-2 receptors with a reduction in IL-2. Increased levels of IL-6, TNF-α and IFN-γ are found in serum and CSF with increasing severity of the disease. Evidence of peripheral T-cell activation in the form of increased expression of HLA-DR, membrane-bound IL-2 receptor and transferrin receptor are also noticed during the course of GBS.

The presence of auto-antibodies in several patients with GBS (plus its variants) and MMN, suggests that the effect on B-cells and the complement pathway might play a significant role in the therapeutic efficacy of IVIg in these conditions. However not all patients have a clearly detectable antibody and hence T-cells and cytokines are thought to have a significant pathogenic role, as discussed earlier.

In conclusion, there are several possible mechanisms of action of IVIg, none of which has been conclusively proven to be the dominating pathway. The main mechanisms which are likely to be responsible in inflammatory neuropathies are summarised in Box 1. It is possible that combinations of different mechanisms are involved in several autoimmune neurological diseases where IVIg remains a highly effective therapy.

## References

[1] (1984). High-dose intravenous gammaglobulin for myasthenia gravis. Lancet.

[2] (1997). Randomised trial of plasma exchange, intravenous immunoglobulin, and combined treatments in Guillain-Barre syndrome. Plasma Exchange/Sandoglobulin Guillain-Barre Syndrome Trial Group. Lancet.

[3] Aukrust P, Froland SS, Liabakk NB, Muller F, Nordoy I, Haug C, Espevik T (1994). Release of cytokines, soluble cytokine receptors, and interleukin-1 receptor antagonist after intravenous immunoglobulin administration *in vivo*. Blood.

[4] Azulay JP, Blin O, Pouget J, Boucraut J, Bille-Turc F, Carles G, Serratrice G (1994). Intravenous immunoglobulin treatment in patients with motor neuron syndromes associated with anti-GM1 antibodies: a double-blind, placebo-controlled study. Neurology.

[5] Basta M, Illa I, Dalakas MC (1996). Increased in vitro uptake of the complement C3b in the serum of patients with Guillain-Barre syndrome, myasthenia gravis and dermatomyositis. J. Neuroimmunol.

[6] Basta M, Van Goor F, Luccioli S, Billings EM, Vortmeyer AO, Baranyi L, Szebeni J, Alving CR, Carroll MC, Berkower I, Stojilkovic SS, Metcalfe DD (2003). F(ab)'2-mediated neutralization of C3a and C5a anaphylatoxins: a novel effector function of immunoglobulins. Nat. Med.

[7] Blank M, Anafi L, Zandman-Goddard G, Krause I, Goldman S, Shalev E, Cervera R, Font J, Fridkin M, Thiesen HJ, Shoenfeld Y (2007). The efficacy of specific IVIG anti-idiotypic antibodies in antiphospholipid syndrome (APS): trophoblast invasiveness and APS animal model. Int. Immunol.

[8] Bouhlal H, Hocini H, Quillent-Gregoire C, Donkova V, Rose S, Amara A, Longhi R, Haeffner-Cavaillon N, Beretta A, Kaveri SV, Kazatchkine MD (2001). Antibodies to C-C chemokine receptor 5 in normal human IgG block infection of macrophages and lymphocytes with primary R5-tropic strains of HIV-1. J. Immunol.

[9] Chapel HM (1994). Consensus on diagnosis and management of primary antibody deficiencies. Consensus Panel for the Diagnosis and Management of Primary Antibody Deficiencies.[erratum appears in BMJ 1994 Apr 2;308(6933):913]. BMJ.

[10] Chapel HM, Spickett GP, Ericson D, Engl W, Eibl MM, Bjorkander J (2000). The comparison of the efficacy and safety of intravenous versus subcutaneous immunoglobulin replacement therapy. J. Clin. Immunol.

[11] Creange A, Gregson NA, Hughes RA (2003). Intravenous immunoglobulin modulates lymphocyte CD54 and monocyte FcgammaRII expression in patients with chronic inflammatory neuropathies. J. Neuroimmunol.

[12] Crow AR, Song S, Semple JW, Freedman J, Lazarus AH (2007). A role for IL-1 receptor antagonist or other cytokines in the acute therapeutic effects of IVIg?. Blood.

[13] Dalakas MC (2002). Mechanisms of action of IVIg and therapeutic considerations in the treatment of acute and chronic demyelinating neuropathies. Neurology.

[14] Dalakas MC (2004). The use of intravenous immunoglobulin in the treatment of autoimmune neuromuscular diseases: evidence-based indications and safety profile. Pharmacol. Ther.

[15] Dalakas MC, Quarles RH, Farrer RG, Dambrosia J, Soueidan S, Stein DP, Cupler E, Sekul EA, Otero C (1996). A controlled study of intravenous immunoglobulin in demyelinating neuropathy with IgM gammopathy. Ann. Neurol.

[16] Devathasan G, Kueh YK, Chong PN (1984). High-dose intravenous gammaglobulin for myasthenia gravis. Lancet.

[17] Dietrich G, Kazatchkine MD (1990). Normal immunoglobulin G (IgG) for therapeutic use (intravenous Ig) contain antiidiotypic specificities against an immunodominant, disease-associated, cross-reactive idiotype of human anti-thyroglobulin autoantibodies. J. Clin. Investig.

[18] Fateh-Moghadam A, Wick M, Besinger U, Geursen RG (1984). High-dose intravenous gammaglobulin for myasthenia gravis. Lancet.

[19] Federico P, Zochodne DW, Hahn AF, Brown WF, Feasby TE (2000). Multifocal motor neuropathy improved by IVIg: randomized, double-blind, placebo-controlled study. Neurology.

[20] Frank MM, Basta M, Fries LF (1992). The effects of intravenous immune globulin on complement-dependent immune damage of cells and tissues. Clin. Immunol. Immunopathol.

[21] Grosse-Wilde H, Blasczyk R, Westhoff U (1992). Soluble HLA class I and class II concentrations in commercial immunoglobulin preparations. Tissue Antigens.

[22] Halstead SK, Humphreys PD, Zitman FM, Hamer J, Plomp JJ, Willison HJ (2008). C5 inhibitor rEV576 protects against neural injury in an in vitro mouse model of Miller Fisher syndrome. J. Peripher. Nerv. Syst.

[23] Halstead SK, Zitman FM, Humphreys PD, Greenshields K, Verschuuren JJ, Jacobs BC, Rother RP, Plomp JJ, Willison HJ (2008). Eculizumab prevents anti-ganglioside antibody-mediated neuropathy in a murine model. Brain.

[24] Harbo T, Andersen H, Hess A, Hansen K, Sindrup SH, Jakobsen J (2009). Subcutaneous versus intravenous immunoglobulin in multifocal motor neuropathy: a randomized, single-blinded cross-over trial. Eur. J. Neurol.

[25] Hughes RA, Allen D, Makowska A, Gregson NA (2006). Pathogenesis of chronic inflammatory demyelinating polyradiculoneuropathy. J. Peripher. Nerv. Syst.

[26] Hughes RA, Donofrio P, Bril V, Dalakas MC, Deng C, Hanna K, Hartung HP, Latov N, Merkies IS, van Doorn PA (2008). Intravenous immune globulin (10% caprylate-chromatography purified) for the treatment of chronic inflammatory demyelinating polyradiculoneuropathy (ICE study): a randomised placebo-controlled trial. Lancet Neurol.

[27] Hughes RA, Raphael JC, Swan AV, van Doorn PA (2001). Intravenous immunoglobulin for Guillain-Barre syndrome. Cochrane Database Syst. Rev.

[28] Hurez V, Kaveri SV, Mouhoub A, Dietrich G, Mani JC, Klatzmann D, Kazatchkine MD (1994). Anti-CD4 activity of normal human immunoglobulin G for therapeutic use. (Intravenous immunoglobulin, IVIg). Therap. Immunol.

[29] Imbach P, Barandun S, d'Apuzzo V, Baumgartner C, Hirt A, Morell A, Rossi E, Schoni M, Vest M, Wagner HP (1981). High-dose intravenous gammaglobulin for idiopathic thrombocytopenic purpura in childhood. Lancet.

[30] Kaneko Y, Nimmerjahn F, Ravetch JV (2006). Anti-inflammatory activity of immunoglobulin G resulting from Fc sialylation. Science.

[31] Kazatchkine MD, Kaveri SV (2001). Immunomodulation of autoimmune and inflammatory diseases with intravenous immune globulin. N. Engl. J. Med.

[32] Kleyweg RP, van der Meche FG, Meulstee J (1988). Treatment of Guillain-Barre syndrome with high-dose gammaglobulin. Neurology.

[33] Kondo N, Kasahara K, Kameyama T, Suzuki Y, Shimozawa N, Tomatsu S, Nakashima Y, Hori T, Yamagishi A, Ogawa T (1994). Intravenous immunoglobulins suppress immunoglobulin productions by suppressing Ca(2+)-dependent signal transduction through Fc gamma receptors in B lymphocytes. Scand. J. Immunol.

[34] Lapointe BM, Herx LM, Gill V, Metz LM, Kubes P (2004). IVIg therapy in brain inflammation: etiology-dependent differential effects on leucocyte recruitment. Brain.

[35] Lee DH, Linker RA, Paulus W, Schneider-Gold C, Chan A, Gold R (2008). Subcutaneous immunoglobulin infusion: a new therapeutic option in chronic inflammatory demyelinating polyneuropathy. Muscle Nerve.

[36] Leger JM, Chassande B, Musset L, Meininger V, Bouche P, Baumann N (2001). Intravenous immunoglobulin therapy in multifocal motor neuropathy: a double-blind, placebo-controlled study. Brain.

[37] Leung DY, Cotran RS, Kurt-Jones E, Burns JC, Newburger JW, Pober JS (1989). Endothelial cell activation and high interleukin-1 secretion in the pathogenesis of acute Kawasaki disease. Lancet.

[38] Lutz HU, Stammler P, Jelezarova E, Nater M, Spath PJ (1996). High doses of immunoglobulin G attenuate immune aggregate-mediated complement activation by enhancing physiologic cleavage of C3b in C3bn-IgG complexes. Blood.

[39] Malik U, Oleksowicz L, Latov N, Cardo LJ (1996). Intravenous gamma-globulin inhibits binding of anti-GM1 to its target antigen. Ann. Neurol.

[40] Marchalonis JJ, Kaymaz H, Dedeoglu F, Schluter SF, Yocum DE, Edmundson AB (1992). Human autoantibodies reactive with synthetic autoantigens from T-cell receptor beta chain. Proc. Natl. Acad. Sci. U.S.A.

[41] Mehta YS, Badakere SS (1996). *In-vitro* inhibition of antiplatelet autoantibodies by intravenous immunoglobulins and Rh immunoglobulins. J. Postgraduate Med.

[42] Mendell JR, Barohn RJ, Freimer ML, Kissel JT, King W, Nagaraja HN, Rice R, Campbell WW, Donofrio PD, Jackson CE, Lewis RA, Shy M, Simpson DM, Parry GJ, Rivner MH, Thornton CA, Bromberg MB, Tandan R, Harati Y, Giuliani MJ (2001). Randomized controlled trial of IVIg in untreated chronic inflammatory demyelinating polyradiculoneuropathy. Neurology.

[43] Mollnes TE, Hogasen K, Hoaas BF, Michaelsen TE, Garred P, Harboe M (1995). Inhibition of complement-mediated red cell lysis by immunoglobulins is dependent on the IG isotype and its C1 binding properties. Scand. J. Immunol.

[44] Nimmerjahn F, Anthony RM, Ravetch JV (2007). Agalactosylated IgG antibodies depend on cellular Fc receptors for *in vivo* activity. Proc. Natl. Acad. Sci. U. S. A.

[45] Nimmerjahn F, Ravetch JV (2008). Anti-inflammatory actions of intravenous immunoglobulin. Annu. Rev. Immunol.

[46] Nobile-Orazio E, Terenghi F (2005). IVIg in idiopathic autoimmune neuropathies: analysis in the light of the latest results. J. Neurol.

[47] Pashov A, Kaveri A, Kazatchkine MD, Bellon B, Kazatchkine MD, Morell A (1996). Suppression of experimental autoimmune encephalomyelitis by intravenous immunoglobulin. Intravenous immunoglobulin: research and therapy.

[48] Rajabally YA, Seow H, Wilson P (2006). Dose of intravenous immunoglobulins in chronic inflammatory demyelinating polyneuropathy. J. Peripher. Nerv. Syst.

[49] Raphael JC, Chevret S, Harboun M, Jars-Guincestre MC (2001). Intravenous immune globulins in patients with Guillain-Barre syndrome and contraindications to plasma exchange: 3 days versus 6 days. J. Neurol. Neurosurg. Psychiatry.

[50] Raphael JC, Chevret S, Hughes RA, Annane D (2002). Plasma exchange for Guillain-Barre syndrome. Cochrane Database Syst. Rev.

[51] Rossi F, Jayne DR, Lockwood CM, Kazatchkine MD (1991). Anti-idiotypes against anti-neutrophil cytoplasmic antigen autoantibodies in normal human polyspecific IgG for therapeutic use and in the remission sera of patients with systemic vasculitis. Clin. Exp. Immunol.

[52] Rossi F, Kazatchkine MD (1989). Antiidiotypes against autoantibodies in pooled normal human polyspecific Ig. J. Immunol.

[53] Rossi F, Sultan Y, Kazatchkine MD (1988). Anti-idiotypes against autoantibodies and alloantibodies to VIII:C (anti-haemophilic factor) are present in therapeutic polyspecific normal immunoglobulins. Clin. Exp. Immunol.

[54] Samuelsson A, Towers TL, Ravetch JV (2001). Anti-inflammatory activity of IVIG mediated through the inhibitory Fc receptor. Science.

[55] Saoudi A, Hurez V, de Kozak Y, Kuhn J, Kaveri SV, Kazatchkine MD, Druet P, Bellon B (1993). Human immunoglobulin preparations for intravenous use prevent experimental autoimmune uveoretinitis. Int. Immunol.

[56] Schuller E, Govaerts A (1983). First results of immunotherapy with immunoglobulin G in multiple sclerosis patients. Eur. Neurol.

[57] Sharief MK, Ingram DA, Swash M, Thompson EJ (1999). I.v. immunoglobulin reduces circulating proinflammatory cytokines in Guillain-Barre syndrome. Neurology.

[58] Silvestris F, D'Amore O, Cafforio P, Savino L, Dammacco F (1996). Intravenous immune globulin therapy of lupus nephritis: use of pathogenic anti-DNA-reactive IgG. Clin. Exp. Immunol.

[59] Stangel M, Schumacher HC, Ruprecht K, Boegner F, Marx P (1997). Immunoglobulins for intravenous use inhibit TNF alpha cytotoxicity *in vitro*. Immunol. Invest.

[60] Stohl W, Elliot JE (1996). *In vitro* inhibition by intravenous immunoglobulin of human T cell-dependent B cell differentiation induced by staphylococcal superantigens. Clin. Immunol. Immunopathol.

[61] Sultan Y, Kazatchkine MD, Maisonneuve P, Nydegger UE (1984). Anti-idiotypic suppression of autoantibodies to factor VIII (antihaemophilic factor) by high-dose intravenous gammaglobulin. Lancet.

[62] Sundblad A, Marcos MA, Malanchere E, Castro A, Haury M, Huetz F, Nobrega A, Freitas A, Coutinho A (1994). Observations on the mode of action of normal immunoglobulin at high doses. Immunol. Rev.

[63] Toungouz M, Denys CH, De Groote D, Dupont E (1995). *In vitro* inhibition of tumour necrosis factor-alpha and interleukin-6 production by intravenous immunoglobulins. Br. J. Haematol.

[64] Toyoda M, Pao A, Petrosian A, Jordan SC (2003). Pooled human gammaglobulin modulates surface molecule expression and induces apoptosis in human B cells. Am. J. Transplant.

[65] Van Asseldonk JT, Franssen H, Van den Berg-Vos RM, Wokke JH, Van den Berg LH (2005). Multifocal motor neuropathy. Lancet Neurol.

[66] Van den Berg LH, Kerkhoff H, Oey PL, Franssen H, Mollee I, Vermeulen M, Jennekens FG, Wokke JH (1995). Treatment of multifocal motor neuropathy with high dose intravenous immunoglobulins: a double blind, placebo controlled study. J. Neurol. Neurosurg. Psychiatry.

[67] van Doorn PA, Ruts L, Jacobs BC (2008). Clinical features, pathogenesis, and treatment of Guillain-Barre syndrome. Lancet Neurol.

[68] van Engelen BG, Miller DJ, Pavelko KD, Hommes OR, Rodriguez M (1994). Promotion of remyelination by polyclonal immunoglobulin in Theiler's virus-induced demyelination and in multiple sclerosis. J. Neurol. Neurosurg. Psychiatry.

[69] Vassilev T, Gelin C, Kaveri SV, Zilber MT, Boumsell L, Kazatchkine MD (1993). Antibodies to the CD5 molecule in normal human immunoglobulins for therapeutic use (intravenous immunoglobulins, IVIg). Clin. Exp. Immunol.

[70] Vassilev T, Yamamoto M, Aissaoui A, Bonnin E, Berrih-Aknin S, Kazatchkine MD, Kaveri SV (1999). Normal human immunoglobulin suppresses experimental myasthenia gravis in SCID mice. Eur. J. Immunol.

[71] Vassilev TL, Kazatchkine MD, Van Huyen JP, Mekrache M, Bonnin E, Mani JC, Lecroubier C, Korinth D, Baruch D, Schriever F, Kaveri SV (1999). Inhibition of cell adhesion by antibodies to Arg-Gly-Asp (RGD) in normal immunoglobulin for therapeutic use (intravenous immunoglobulin, IVIg). Blood.

[72] Vermeulen M, van der Meche FG, Speelman JD, Weber A, Busch HF (1985). Plasma and gamma-globulin infusion in chronic inflammatory polyneuropathy. J. Neurol. Sci.

[73] Viard I, Wehrli P, Bullani R, Schneider P, Holler N, Salomon D, Hunziker T, Saurat JH, Tschopp J, French LE (1998). Inhibition of toxic epidermal necrolysis by blockade of CD95 with human intravenous immunoglobulin. Science.

[74] Willison HJ, Halstead SK, Beveridge E, Zitman FM, Greenshields KN, Morgan BP, Plomp JJ (2008). The role of complement and complement regulators in mediating motor nerve terminal injury in murine models of Guillain-Barre syndrome. J. Neuroimmunol.

[75] Xu C, Poirier B, Van Huyen JP, Lucchiari N, Michel O, Chevalier J, Kaveri S (1998). Modulation of endothelial cell function by normal polyspecific human intravenous immunoglobulins: a possible mechanism of action in vascular diseases. Am. J. Pathol.

[76] Yu Z, Lennon VA (1999). Mechanism of intravenous immune globulin therapy in antibody-mediated autoimmune diseases. N. Engl. J. Med.

[77] Zweiman B (1989). Theoretical mechanisms by which immunoglobulin therapy might benefit myasthenia gravis. Clin. Immunol. Immunopathol.

